# Experiences of a Digital Mental Health Intervention from the Perspectives of Young People Recovering from First-Episode Psychosis: A Focus Group Study

**DOI:** 10.3390/ijerph20095745

**Published:** 2023-05-08

**Authors:** Shalini Lal, Ryan Tobin, Stephanie Tremblay, John F. M. Gleeson, Simon D’Alfonso, Geraldine Etienne, Ridha Joober, Martin Lepage, Mario Alvarez-Jimenez

**Affiliations:** 1 School of Rehabilitation, Faculty of Medicine, University of Montréal, Montréal, QC H3C 3J7, Canada; 2 Youth Mental Health and Technology Lab, Health Innovation and Evaluation Hub, CHUM Research Centre, Montréal, QC H2X 0A9, Canada; ryan.tobin2@mail.mcgill.ca (R.T.); stephanie.tremblay2@mail.mcgill.ca (S.T.); geraldine.etienne@mail.mcgill.ca (G.E.); 3 Prevention and Early Intervention Program for Psychosis, Douglas Mental Health University Institute, Montréal, QC H4H 1R3, Canada; ridha.joober@mcgill.ca (R.J.); martin.lepage@mcgill.ca (M.L.); 4 School of Physical and Occupational Therapy, McGill University, Montréal, QC H3G 1Y5, Canada; 5 Healthy Brain and Mind Research Centre, School of Behavioural and Health Sciences, Australian Catholic University, Fitzroy 3065, Australia; john.gleeson@acu.edu.au; 6 School of Computing and Information Systems, University of Melbourne, Parkville 3010, Australia; dalfonso@unimelb.edu.au; 7 Orygen, Parkville 3052, Australia; mario.alvarez@orygen.org.au; 8 Department of Psychiatry, McGill University, Montréal, QC H3A 1A1, Canada; 9 Centre for Youth Mental Health, University of Melbourne, Parkville 3052, Australia

**Keywords:** psychotic disorders, mental health, telemedicine, young adult, mental health services, e-mental health, virtual care, schizophrenia, Horyzons, digital health innovation

## Abstract

Horyzons is a digital health intervention designed to support recovery in young people receiving specialized early intervention services for first-episode psychosis (FEP). Horyzons was developed in Australia and adapted for implementation in Canada based on input from clinicians and patients (Horyzons–Canada Phase 1) and subsequently pilot-tested with 20 young people with FEP (Horyzons–Canada Phase 2). Objective: To understand the experiences of young adults with FEP who participated in the pilot study based on focus group data. Methods: Among the twenty individuals that accessed the intervention, nine participated across four focus groups. Three team members were involved in data management and analysis, informed by a thematic analysis approach. A coding framework was created by adapting the Phase 1 framework to current study objectives, then revised iteratively by applying it to the current data. Once the coding framework was finalized, it was systematically applied to the entire dataset. Results: Four themes were identified: (1) Perceiving Horyzons-Canada as helpful for recovery; (2) Appreciating core intervention components (i.e., peer networking; therapeutic content; moderation) and ease of use; (3) Being unaware of its features; and (4) Expressing concerns, suggestions, and future directions. Conclusions: Horyzons-Canada was well received, with participants wanting it to grow in scale, accessibility, and functionality.

## 1. Introduction

Psychotic disorders have a median lifetime prevalence of 0.75%, and although considered low-prevalence disorders, in terms of burden of disease, they are associated with the leading causes of disability worldwide [1,2]. Despite the development of specialized early interventions (SEI), relapse and service disengagement rates remain high, and improvements in symptoms and global functioning often fade after the first two years of treatment [3,4,5,6]. Moreover, with specialized services occurring mostly in person, some patients develop a preference for less intensive forms of follow-up over time [7]. These factors highlight a need for new, accessible, sustainable, and effective models of service delivery for youth with first-episode psychosis (FEP). Information and communication technologies (ICTs) provide a potential pathway for addressing these needs [8,9,10,11]. One digital health innovation that has shown promising results in youth recovering from psychosis is Horyzons, a web-based, moderated online social therapy (MOST) intervention designed to sustain treatment benefits of SEI and promote long-term social functioning [12,13].

### 1.1. Overview of Horyzons

Horyzons was developed iteratively over 30 months with the collaboration of patients and a multidisciplinary team. This intervention delivers evidence- and strengths-based targeted psychosocial interventions, which are supported by a moderated online social networking environment. In terms of therapy content, two main activities exist on the platform. “Take a Step” activities are interactive therapy modules designed to develop a multitude of psychological skills. Additionally, the platform offers over 350 interactive behavioral activities, known as “Actions” or “Do Its”, helping participants apply mindfulness, self-compassion, and their strengths in real-world situations. Regarding peer-to-peer web-based social networking, users can contribute posts and comments, share experiences, and give and receive social support through a Facebook-style newsfeed, called “The Café” [14]. Another social networking feature, known as “Talk It Out”, allows users to nominate problems or difficulties that they would like to discuss, brainstorm, and try out possible solutions with other users and website moderators. Lastly, referred to as clinical and peer moderators, clinicians and peer support workers offer moderation (i.e., sharing their expertise and support) to participants on the platform, respectively. Clinical moderators provide guidance, oversee safety on the platform and tailor content to each participant’s clinical needs. Peer moderators are trained peer support workers with previous mental health difficulties who assist with participant orientation, provide support, and foster engagement on the platform.

Horyzons was first pilot tested with a sample of 20 Australian young adults for its feasibility, acceptability, utility, and safety [12]. Over one month, no dropouts or incidents were identified, while 70% of participants engaged highly with the platform for at least three weeks. More recently, a randomized controlled trial (RCT) study by Alvarez-Jimenez et al. [15] reported that, compared to treatment as usual, Horyzons users had significantly higher vocational/educational attainment, fewer visits to emergency services, and lower hospitalization rates at follow-up. MOST (the platform that Horyzons is based on) has now been successfully adapted and piloted in a group of young people at ultra-high risk for psychosis [16], depression [17], and with help-seeking young people [18]. In addition to the accumulating quantitative evidence supporting Horyzons as a beneficial intervention, a handful of studies have used qualitative methodologies to explore the perspectives and experiences of participants using the platform.

To complement quantitative support for the first pilot study on Horyzons [12], Lederman et al. [13] interviewed study participants about their experience using Horyzons. In this qualitative study, users reported that Horyzons provided them with a greater sense of belonging, safety, and security within the group, greater insight into their condition, increased positive thoughts, accountability, and individual strengths, and found the platform visually appealing and engaging to use.

Subsequently, Valentine et al. [19] interviewed twelve participants across platform engagement levels (i.e., very low, low, moderate, high usage) who partook in the Horyzons RCT study [15]. Overall, participants felt that commonly shared lived experiences catalyzed the removal of social barriers and facilitated connection between participants within the online social network. Secondly, participants felt they benefitted from both receiving and providing online support to people that could relate to their difficulties. Third, Horyzons’ upbeat environment was welcoming and a contributing factor to high engagement for some, while others found that it prevented sharing of negative experiences and difficult topics. Lastly, participants identified amotivation in using the social networking feature, paranoia related to dissemination of personal information, and sharing anxiety as factors that inhibited engagement.

Using the same dataset, Valentine et al. [20] found that individual Horyzons participants often experienced online therapy quite differently. Participants described Horyzons therapy content as a way to seek help on demand and distract themselves from difficult thoughts and experiences. Next, participants found therapy content applicable to everyday life and helpful for normalizing their mental health difficulties. Furthermore, some described therapy content as a revision of previous knowledge learned in therapy, which was helpful for some and redundant for others. Lastly, the self-directed nature of therapy empowered some participants to feel in control of their mental health treatment journey, whereas others felt overwhelmed by the level of independence.

### 1.2. Horyzons–Canada (HoryzonsCa)

Although research has explored users’ experiences on a live Horyzons platform, no study has explored the opinions and experiences of an adapted live version of Horyzons tailored specifically to young people with FEP living in other cultural, healthcare, and geographical settings. Looking to implement Horyzons to fit the Canadian culture and healthcare context, Lal and colleagues [21] conducted an adaptation study where 15 clinicians and 11 patients living in Canada provided feedback on Horyzons’ therapy content and layout. Through focus groups, interviews, and consultations, participants (i.e., clinicians and patients) expressed appreciation for HoryzonsCa’s therapeutic approach and found it relatable for young people. Some participants appreciated the platform’s calm and neutral look, whereas others preferred a more colorful and dynamic experience. Several clinicians raised concerns regarding patient safety on The Café (e.g., mutual respect), application of clinical moderation (e.g., crisis response management and integration of moderation into clinical workflow), and internet connectivity in rural communities. Taking these perspectives into consideration, adaptations were made to the HoryzonsCa platform to better fit local needs and prepare it for a pilot study of the intervention. The pilot study aimed to investigate the acceptability, safety, and potential efficacy of a live version of the HoryzonsCa intervention among 20 participants over 8 weeks. Unlike the previous adaptation study, the pilot study offered participants access to moderation and the ability to freely communicate with other participants. Further details on the pilot study have been published in a protocol paper including additional information on HoryzonsCa and its study methods [22], and the pilot study has been registered as a trial in the ISRCTN Registry (HoryzonsCa Phase 2 Pilot Study Trial Registration: ISRCTN43182105).

### 1.3. Aims

The current exploratory study aims to understand the experiences and opinions of HoryzonsCa pilot study participants through a thematic analysis of focus group data.

## 2. Methods

### 2.1. Participants and Study Design

This study took place in an urban FEP clinic in Montreal, Canada. This clinic targets 14- to 35-year-olds with a diagnosis of affective or non-affective psychosis, who have had no more than 1 month’s previous antipsychotic treatment, do not have organic brain damage, a pervasive developmental disorder, an IQ below 70, or epilepsy, and do not have substance-induced psychosis. A comorbid diagnosis of substance abuse is not an exclusion criterion for access to the program. To meet the inclusion criteria specifically for the pilot study, participants must have been 18 years or older, diagnosed with a psychotic disorder, and have been receiving specialized services for first-episode psychosis, and within their first 3 years of treatment. Further, participants must have been considered stable, at low or moderate suicidal risk for the month preceding the study, and capable of participating in a focus group (as judged by their primary clinicians). Participants were excluded if they had an intellectual disability or were diagnosed with antisocial or borderline personality disorders, were hospitalized at the time of recruitment, were in the acute phase of mania or psychosis, or were unable to speak or read English. Twenty-three participants were recruited for HoryzonsCa Phase 2 between the 17th of September 2018 and the 1st of June 2019. Of these, 20 participants were given access to the intervention and were later invited to take part in focus group sessions. In total, nine agreed to participate in focus group sessions, with an average attendance of 3.5 participants at each focus group. Of the nine participants, two attended focus groups twice and one participated three times in the interest of collecting as much HoryzonsCa feedback as possible over time. The current sample comprises six females, two males, and one person who identified with neither. The current sample ranges from 18 to 34 years old with a mean age of 26.9 years old (SD = 6.2). Participants represented a range of single and mixed ethnicities, including of Latin American, European, African, Indigenous, and Asian descent. Five participants were college or university educated, and four held a high school diploma. Participants had access to the HoryzonsCa platform for at least 1 month before participating in a focus group. There were no significant differences in sociodemographic characteristics between participants who attended the focus groups (n = 9) and those who did not (n = 14). Within the sample of participants who accessed the platform, when comparing the website usage analytics of participants who attended the focus groups (n = 9) with those who did not (n = 11), we found significant differences in the frequency of logins; that is, individuals who participated in the focus groups (M = 11.78, SD = 8.36) used the intervention more frequently than those who did not (M = 3.27, SD = 3.1), t(18) = 3.14, *p* = 0.01.

### 2.2. Data Collection

Four focus groups were conducted between November 2018 and April 2019. On average, discussions lasted 1.5 h and were run by two focus group facilitators, the primary author (A1) and a research assistant (A6). The primary author (female), who served as the lead focus group facilitator, has extensive previous experience in focus group facilitation. The primary author’s research program is focused on the acceptability and use of technology to improve access to and the quality of mental health services. The research assistant (female) has extensive experience in conducting individual interviews with this clinical population. Neither focus group facilitator had a previous clinical relationship with participants. Additionally, the focus group facilitators were transparent about the goal of data collection with participants. Focus groups were conducted in person in an FEP clinic conference room. The focus group topic guide was adapted based on the HoryzonsCa Phase 1 [21] script to include questions that better captured users’ experiences of a live HoryzonsCa platform. Focus group topics included general impressions of HoryzonsCa (e.g., likes and dislikes), its ease of use, its utility towards well-being, how HoryzonsCa could be improved and any other suggestion for intervention implementation and evaluation. Additional focus group guide details can be found on the Phase 1 adaptation protocol [21]. Though focus groups were conducted primarily in English, occasionally some participants for whom French was their primary language expressed themselves in French. As such, the focus group facilitators would translate or summarize, when needed, to ensure group comprehension. Ethics approval was obtained from the Research Ethics Board of the Centre intégré universitaire de santé et de services sociaux de l’Ouest-de-l’Île-de-Montréal on the 11th of April 2018 (#IUSMD 17-54). Participants provided informed and written consent before participating in the study.

### 2.3. Data Management and Analysis

Focus groups were audio recorded and manually transcribed verbatim. Transcriptions were mainly completed by two research assistants (A2 and A3) supervised by the primary author, all of whom are fluently bilingual. Each transcript was validated by a second member of the team and finalized by the primary author. Transcripts were not returned to participants for comment or correction. Qualitative data were managed using ATLAS.ti (version 22; ATLAS.ti Scientific Software Development GmbH) and analyzed using a deductive and inductive approach informed by Braun and Clarke’s six phases of thematic analysis [23]. The first phase of thematic analysis requires the researchers to become familiar with the data by transcribing verbal data into written form. In the second step, researchers generate initial codes and apply them to their dataset, which is then followed by searching for key themes in the third phase. The fourth phase involves reviewing themes by either grouping, splitting, or deleting existing themes. The fifth phase requires identifying the essence of each theme and what aspect of the data captures each theme. Lastly, phase six entails report writing of fully worked-out themes.

Once all focus groups were transcribed, transcribers (A2 and A3) individually reviewed and summarized each focus group to familiarize themselves with the content. These summaries were discussed by the analysis team (A1, A2 and A3). Using the coding framework from HoryzonsCa Phase 1 as a template [21], a preliminary coding framework was created based on the current study objectives, patterns in the initial coding of the transcripts, and team discussion. Next, research assistants (A2 and A3) applied the revised coding framework to one focus group to assess how accurately it represented the data. The analysis team then met to discuss how the coding framework could be improved, followed by testing the relevancy of the revised framework to additional data, an iterative process that occurred over several cycles, resulting in a finalized coding framework (see Table S1 in the Supplementary File). Once the coding framework was finalized, the research assistants (A2 and A3) then coded one focus group independently, and the discrepancies were discussed and reviewed with the primary author to establish reliability across coders. For the remaining focus groups, one research assistant (A2) was assigned to a focus group, while the other validated the coding (A3). These validation processes revealed only minor discrepancies, which were discussed among the research team to achieve consensus. After the coding was finalized, one research assistant (A2) identified all codes mentioned by a minimum of three individual participants, the quotes of which were then analyzed and discussed by the team to identify large overarching themes. The team set the threshold of including themes identified by at least three separate participants to avoid overrepresenting the opinions and experiences of those who attended multiple focus groups. Lastly, sub-themes were identified within each theme to better understand the nuances within a specific topic. The participants were not re-contacted to provide feedback on findings.

## 3. Results

The thematic analysis of focus group data resulted in four core themes (See Table 1): (1) Perceiving HoryzonsCa as helpful for recovery; (2) Appreciating core intervention components (e.g., peer networking; therapeutic content; moderation) and ease of use; (3) Being unaware of Horyzons’ features; and (4) Expressing concerns, suggestions, and future directions, which we will describe below. Quotes are labelled with ID codes (e.g., P1), and illustrative quotes are drawn from all nine participants. Gender has been removed from the ID codes to further protect the anonymity of participants. Additionally, the term “Facilitator” has been used in quotes to identify members of the research team who conducted the focus groups. Utterances, such as “uh”, and repetitive words, such as “to to to”, have been removed from quotations to facilitate reading. Quotes in French were translated to English by a research assistant and validated by another team member using back translation.

### 3.1. Perceiving HoryzonsCa as Helpful for Recovery: “I Can Apply It to My Real-Life Situations”

The majority of participants (8/9, 89%) stated that HoryzonsCa was helpful for their recovery and daily life. There were four sub-themes identified in this theme. First, participants (n = 4) expressed that HoryzonsCa was effective in helping to cope with stress and anxiety, for example, by offering strategies:

For me it’s more like strategies… it offers great insight, and uhhh… you know the examples are not always what I’m facing, but I can apply it to my real-life situations. It helps.

[P5]

Second, participants (n = 3) felt that HoryzonsCa helped them focus on self-improvement and feel more self-connected:

The first thing I like the most is that it allows us to develop our qualities… like perseverance, courage.

[P7; Translated]

Third, participants (n = 3) expressed that the tips on HoryzonsCa were applicable to their everyday life and recovery:

Well, there are tools and tips to help you go through life.

[P1]

Fourth, participants (n = 3) shared that HoryzonsCa helped them become better informed about the nature of their illness:

If someone is new or they’re like ok Horyzon [sCa], ok this is an interesting website! And they look at it like: Oh! That’s what I went through! Or oh, this is what I’m going through! … I didn’t even know what psychosis was!

[P2]

### 3.2. Appreciating Core Intervention Components and Ease of Use

Participants commonly expressed satisfaction and appreciation with HoryzonsCa. There were four sub-themes identified in this theme; the first three pertained to the usefulness of core intervention components (i.e., connecting with peers, therapeutic content, and moderation), and the fourth sub-theme was ease of use.

Connecting with Peers: “It’s a community that’s like me”.

Most participants (7/9; 78%) expressed appreciation for the peer networking aspect of the platform; they enjoyed connecting with others who share similar lived experiences, the sense of community centered around mental health, and that it was a place where they could express themselves and feel heard:

It helps me open up in the sense that, like… Sharing, for me it’s something useful.

[P3]

Therapeutic Content: “Not everyone knows where to go to get help”

More than half of the participants (5/9, 56%) expressed an appreciation for the therapeutic content on the platform. Three specifically commented on the reliability and accessibility of helpful, evidence-based information that may otherwise be difficult or time-consuming to find:

If I would have been looking for information about mental health… I can go and see discussions [anywhere] on the internet that says anything… it wouldn’t necessarily help there… it’s [HoryzonsCa] a good place to look for information…

[P4; Translated]

Additionally, participants (n = 3) were impressed with the breadth of content featured on the platform and the possibility to save content for later access. There was a particularly strong positive sentiment towards content delivered using comics, with two participants expressing their enthusiasm about it as they found them engaging and understandable.

Moderation: “They were very present… that was helpful”

Nearly all participants (8/9, 89%) expressed appreciation for moderation on HoryzonsCa. Participants (n = 6) explained that moderators were impactful, helpful, and supportive:

I find it was well!… They were very present getting a text every week, tagging you in posts, that was helpful.

[P6]

Participants (n = 3) found that moderators effectively guided them towards helpful content and appreciated their help with finding employment and navigating the platform:

He gave me a little bit, hmm like a road to follow and help me follow how to find a job and tell me do a little bit of that you will be nice and continue with your exercises and I do it and it help me out… They helped me a lot.

[P3]

Lastly, participants (n = 2) expressed that the moderators became an important part of their treatment team that they could depend on for support:

But this can help. Instead of clogging up the system and then not having anyone to talk to, [my case manager and I] won’t [need to] talk afterwards.

[P4; Translated]

Easy and Familiar to Use: “It’s like Facebook”

More than half of the participants (5/9; 56%) shared that the HoryzonsCa platform was well-designed and easy to navigate:

[P9]: Easy.

[P2]: I found it really simple… I like the layout.

Ease of use was also highlighted in relation to its familiarity with other well-established social media websites, such as Facebook.

### 3.3. Being Unaware of HoryzonsCa’s Features: “I Don’t Think Everybody Knows about It”

Another common theme was that two-thirds (6/9; 67%) of participants were unaware of at least one HoryzonsCa feature. Typically, these features were live chat, profile settings, and communication tools. The live chat was the most frequently discussed feature that participants were unaware of:

Yeah, I think you should talk about that too. About the chat a little bit because I don’t think everybody knows about it either.

[P2]

Of those who were aware of the live chat feature, many refrained from using it, as they believed they were unable to send messages to participants and moderators who were not online (i.e., connected to the platform) at the same time as they were. Other features participants were unaware of were related to adjusting their profile and tools that could be used to communicate on The Café, such as tagging others, using the “I’m just venting” posts, and how to use “Talk it Outs”.

### 3.4. Expressing Concerns, Suggestions, and Future Directions

This theme is organized by concerns and suggestions regarding core intervention components and the design of HoryzonsCa, followed by future directions that participants envision for HoryzonsCa. The sub-themes for concerns and suggestions were: lack of peer interaction, need for deeper content, wanting to connect more, and desire for a more professional design.

Peer Support: “Lack of interaction on the platform”

Some participants (4/9, 44%) expressed concerns regarding peer networking on HoryzonsCa, particularly concerning the lack of interaction on both The Café and the online chat:

It was mostly just staff posting stuff. Nobody really posted stuff so I feel that if it was more interactive or if there were more things to do… I feel like it has more potential to grow.

[P8]

Unlike on social media websites where the user decides who their “friends” or “followers” are, some participants (n = 2) expressed that it was difficult to engage with others that they did not know.

Several participants (4/9, 44%) provided suggestions to improve the peer networking aspect on HoryzonsCa. One popular suggestion was to host a live chat party:

[P1]: We should do a chat party!… Yeah! Just like a topic of discussion, stuff like that.

[Facilitator]: Yes. So there should be like a topic of discussion.

[P3]: We can do a theme each week.

[P4]: Like the theme of the month is, let’s say depression. So people share their tips or experiences or how they overcame it, and then we talk together but maybe not necessarily in The Café. It can be on the live chat but one… with each month it differs.

Other ideas expressed by three participants to increase engagement included reducing posts unrelated to the discussion on The Café (e.g., “Do It” posts, etc.), having weekly themes featured on HoryzonsCa, and encouraging general discussion unrelated to mental health on The Café:

Content: “We need deeper knowledge”

More than half of the participants (5/9, 56%) expressed content-related concerns on HoryzonsCa. One such concern voiced by two participants regarded the way scientific facts and other information were presented.

Further, some participants (n = 3) felt that the content on the platform was not specific enough to their needs, particularly concerning psychosis, or did not have enough activities:

It’s a lot of basic knowledge… You know so all those things like we’ve already learnt it, so we need deeper knowledge… Because anxiety, everybody has anxiety, you just have to deal with it, anxiety is very vague… That’s what I grasped from it. Like if you gave me one word to describe it, it would be how to manage anxiety, breathing exercises, things like that! But like what we’ve been through, psychosis, maybe I shouldn’t speak for the group, but it’s a lot more than anxiety, it’s beyond!

[P6]

Two-thirds of participants (6/9, 67%) offered suggestions to improve the content on HoryzonsCa. In particular, five participants saw great value in the testimonies of those who have lived and recovered from similar mental health experiences.

Participants (n = 2) also suggested making the comics interactive:

What would be good is that you can play the comics… that it’s an interaction, not just reading because I hate reading. So if you can interact with the comics, then you can do one thing or the other or whatever the comics are doing.

[P4, Translated]

Some participants (n = 4) suggested including facts about specific symptoms and concrete steps on how to deal with them:

And maybe more facts… what’s the average relapse? What medications are available? What type of therapy [there] is?

[P6]

Finally, the ability to freely access material without having to complete various sections was another suggestion provided by participants (n = 2).

Moderation: “They could relate to us with their own experience on mental health”

Although participants did not express concerns regarding moderation, many (6/9, 67%) did share suggestions to further improve this aspect of the platform. Three participants felt that they would benefit from hearing the moderators’ own mental health experiences:

Actually, it would be cool if the moderators did the “Do It’s” and then shared what they feel.

[P7]

Further, some participants (n = 3) wanted the moderators to connect with them on a deeper level, for example through a phone chat. Some participants (n = 2) suggested that the moderators could increase engagement on the platform by facilitating more general open-ended discussions on The Café:

Yeah just like once every two weeks. Once a week. Not necessarily in relation to your current movie or your current music… how was your week or uh, what do you want to go do on the weekend?

[P4]

Lastly, some participants (n = 2) stated that they would like to add members of their local mental health treatment team to the HoryzonsCa’s moderation structure:

[P1]: It would be better to have someone who is from [first-episode psychosis program].

[Facilitator]: yes, why? …

[P4]: Let’s say, a manager who is not your case manager. He won’t know you. But at least know how it works.

[Facilitator]: The context.

[P4]: Yeah, the context. That’s it. He knows that.

[P1]: He will know you personally.

Design: “Something more professional”

More than half (5/9, 56%) of the participants proposed ideas on how to improve its original design. One wanted to have a more professional-looking website, while others preferred a website that was more visually attractive. The importance of colors was also brought up by some participants (n = 2) to ensure visual clarity:

Well the website is kinda like, the format is kinda boring… Maybe more colors and like… And art… Like yeah. I don’t know, just different stuff… Just spice it up.

[P8]

Future Directions

Many participants shared how they envision HoryzonsCa developing and being implemented in the future. For example, some participants (3/9, 33%) expressed a desire for a French or more bilingual platform. After language concerns arose in the first two focus groups, it was decided that moderators would post in both English and French, and participants were encouraged to respond to posts and interact on the website in their preferred language. Those who expressed their desire for a French platform showed their appreciation for these changes in subsequent focus groups (i.e., Focus Groups 3 and 4):

Yes, both languages are good because not everyone speaks one or the other.

[P4]

Further, participants were eager to use HoryzonsCa to continue their recovery process and to see it be implemented on a larger scale. Two participants specifically shared that they were interested in using the platform again once it becomes widely adopted and available.

To further increase its impact, one participant suggested that they would like to see community resources begin referring participants to HoryzonsCa. Although several 24/7 community helpline resources are listed on the platform, some participants (n = 3) would like to see HoryzonsCa take on that role and be monitored around the clock to provide immediate support to those in distress:

We have moderators but if we have like, I don’t know, like a number to call on the website if we felt like we were going through another crisis, or like a registered nurse that we can chat to live because it’s not easy when you’re have a psychosis and you’re calling for help. Because you’re having a crisis.

[P6]

Most participants (7/9, 78%) expressed a strong preference for the utility and convenience of accessing HoryzonsCa through a mobile app:

[P6]: Can you create it as an app?

[P3]: Yes, I think the same. It will be easiest with the phone… We can do it many places different, [not] just in the home with the computer, that won’t stop you from continuing wherever you go.

[P4]: Everyone is used to an app… it’s easier to access.

Finally, some participants (n = 3) hoped that SMS messages and alerts would become a more integral part of the HoryzonsCa experience. More specifically, participants would like to receive activity suggestions by SMS, to receive unlimited SMS notification alerts (limit currently set to 10 per week), and to receive daily SMS mental health challenges and messages of encouragement:

[P4]: If there were challenges that we could do other than to quit smoking but not mental health, that’s also interesting there…

[Facilitator]: Challenges but by text message.

[P7]: Like for example a challenge to encourage.

[P4]: Like, “nice day today”, just little words. Yeah, challenges.

[P7]: Like, to decrease people’s negative thoughts… You can say “Challenge today! Find five positive thoughts and write it on the paper”. On the website and then afterwards it’s a “do it”. [Translated]

## 4. Discussion

This study is the first to qualitatively explore the perceptions and experiences of participants using a live version of Horyzons–Canada. Overall, HoryzonsCa was well received. Further, focus group findings support HoryzonsCa’s mission to provide psychoeducation and a platform for participants to feel heard and supported. The findings also highlight areas for improving future implementation of HoryzonsCa.

Participants found HoryzonsCa helpful for developing strategies to cope with stress and anxiety, for providing daily tips for recovery, and for focusing on self-improvement and connecting to oneself, which are all aligned with the content and purpose of the intervention [12]. Our findings complement previous research on the MOST platform conducted in Australia [13,19,20,24].

In addition, participants appreciated connecting with others who have lived similar experiences and felt that HoryzonsCa offered users a place to feel heard. Peer support can play an instrumental role in mental health recovery as it has been linked to improvements in psychiatric symptoms, social functioning, self-efficacy, self-esteem, quality of life, social connectedness, and feelings of belonging [20,25,26,27]. Systematic reviews suggest that peer support can be more effective in promoting engagement than traditional psychotherapy [28], and previous research on Horyzons suggests that sustained engagement with peers on the platform is an important contributor to better outcomes [29].

Participants also appreciated how easily they could access reliable, evidence-based therapy material through HoryzonsCa. Australian users expressed a similar appreciation for “curated knowledge from mental health professionals” instead of relying on what “Google says” [20]. The HoryzonsCa Phase 1 adaptation study found that only 27% (3/11) and 45% (5/11) of participants regularly used the internet and felt at least somewhat comfortable searching the internet for mental health information, respectively [21]. Locating credible online mental health information can be difficult and time-consuming for young people recovering from FEP [30].

Participants found HoryzonsCa easy to use and related its similarity to other well-established social media websites, such as Facebook, concurring with findings in past evaluations of the platform [12,21]. Interestingly, in HoryzonsCa Phase 1 [21], clinicians, but not participants, expressed concerns about navigating the website, citing the amount of text to navigate, the organization, and the exploratory style of the platform as potential barriers. However, users did not raise these concerns neither in Phase I nor in the current live version of HoryzonsCa, and the overall positive experiences of participants navigating Horyzons across studies continues to be encouraging. The discrepancy in navigation concerns between the clinicians and participants emphasizes the importance of assessing perspectives from both stakeholders regarding digital mental health interventions.

Although participants found the HoryzonsCa moderation helpful and supportive, they rarely distinguished between clinical and peer moderators, which may indicate that participants were unaware of, did not understand, or did not consider the differences between moderation types. It is plausible that the roles and activities of clinical and peer moderators were not distinct enough for participants to understand them as separate entities. Understanding available moderation options may help participants effectively use moderators for specific purposes and issues. In addition, the lack of awareness of several HoryzonsCa features suggests that participants may not have always used the intervention to its full capacity. These findings highlight the need to improve the orientation process and to supplement it with strategies to prompt lesser-known features throughout a participant’s involvement. Possible solutions include increasing feature awareness through HoryzonsCa group meet-ups and posting video tutorials on The Café.

Regarding peer networking, some participants believed that The Café and online chat lacked interaction and some individuals had difficulty connecting with others who they did not personally know. The limited interaction may be due in part to the phased approach to recruitment that was implemented in the pilot study, with only 1 to 2 participants recruited to the site at a time. There is a need for a critical mass of participants to be on the site during the active phase of follow-up to generate interactions. Another factor pertains to sharing anxiety (i.e., the phenomenon of having anxiety towards posting content on social media), which was reported in previous research [19,24]. It is plausible that sharing anxiety contributed to low engagement but was not captured in the focus group data, as active focus group participants may be more extraverted and less likely to experience this phenomenon. In addition, unfamiliarity with other participants may have added to participants’ reticence in posting. Unlike traditional social media websites, where the user decides who they connect with, HoryzonsCa’s Café presents a unique challenge where participants might feel pressured to connect and share intimate details with individuals they do not personally know. To increase social connection, participants suggested holding live chat parties, having weekly themes on The Café, and encouraging discussion unrelated to mental health on The Café. Encouraging shyer individuals to connect with moderators may serve as an important jumping point to encourage participants to post on The Café [24]. More research is needed to gain insights on various factors that limit peer interaction on the website (e.g., having a minimum number of participants, sharing anxiety, sharing a common purpose, having clear rules of engagement, etc.).

Some participants expressed concerns related to the therapeutic content, specifically, that the therapeutic content lacked scientific facts, did not address their specific mental health needs (e.g., obsessive compulsive disorder, psychosis, etc.), and did not always provide new information, which concurs with previous studies describing content as “revision” of material previously learned in therapy [20,24]. These findings suggest that there is a need for illness education online, even if this content is typically provided in specialized early intervention programs (e.g., through psychoeducation workshops and communications with clinical teams). To improve the therapeutic content, participants suggested including testimonies from those who have recovered from similar mental health experiences and the ability to adjust their own pathway to freely access content in no particular order. Seeking advice and learning from those with similar experiences are said to help people with serious mental illnesses experience a sense of community and belonging [31]. In the current study, the peer moderator did provide testimonials; however, only during the post-focus group meet-ups themselves. Communicating testimonials via The Café (e.g., via video message) may reach more users and allow them to engage with the content at their own pace. According to the self-determination theory, individuals feel most engaged when their needs for autonomy and competency are met [32].

Regarding moderation, participants suggested that moderators share their mental health experiences, get to know participants on a deeper level, and spark general discussion unrelated to mental health on The Café. These findings are aligned with research suggesting that rapport building is strongly related to greater self-disclosure, cooperation, and affiliation [33]. Considering that digital interventions and in-person therapy yield similar potentials for therapeutic alliance building, one of the most powerful predictors of psychotherapy efficacy, a stronger moderator–participant relationship on HoryzonsCa would likely lead to better participant outcomes [34,35,36].

Lastly, participants shared general recommendations based on how they envisioned HoryzonsCa developing over time, which overall indicates that they would like it to expand and become more present in their lives by including a greater range of options and features that vary in language, culture, and lifestyle. It is expected that a greater number of participants would be more engaged with a platform that meets the autonomy and competency needs of its users by providing options that fit a wide range of preferences [32].

## 5. Limitations

This is the first study to examine the experiences and perceptions of HoryzonsCa users, and among the first of the international (relative to Australia) Horyzons (and MOST platform) studies to examine users’ perspectives in-depth; however, there are limitations to consider. First, the generalizability of the results is limited given that the sample comprises English-speaking participants living in Quebec. As such, future research on HoryzonsCa delivered in French is needed, which will require the full-scale translation of the content, intervention materials, and moderator training and communication. In addition, future research is also needed on the implementation of HoryzonsCa in multiple sites across Canada. Secondly, the experiences of some participants could be overrepresented in the current study as several attended more than one focus group. Similarly, the current study may strongly reflect the opinions of extroverted individuals who are comfortable in social settings. The comparison of groups in terms of website usage analytics indicates that participants that attended the focus groups were more active users of the platform than those that elected not to attend the groups, indicating a possible self-selection bias. Although overarching themes were identified using opinions and experiences voiced by at least three participants, this methodology does not protect against the same select few representing the opinions of the total sample. To avoid the overrepresentation of the most engaged participants and of those who have attended multiple focus groups, it would be worthwhile to additionally explore the experiences of all HoryzonsCa participants using one-on-one qualitative interviews to capture a broader range of opinions more accurately. Finally, with regards to saturation, which can be sought during data collection, during data analysis, or during both phases [37], in this study, we were only able to address saturation during analysis, as our pool of participants was limited to the 9 from the larger pilot study of 23, who agreed to attend the focus groups. Nonetheless, our coding and categorization of the data continued iteratively until no new categories emerged [37] from coding the transcripts and the three members of the analysis team determined that the set of categories comprehensively represents the data pertaining to the study questions. However, it is possible that with more focus groups or more participants, new categories and themes could be identified. As such, future qualitative research is needed among a greater and more diverse group of individuals to ascertain a more comprehensive range of the opinions and experiences of HoryzonsCa users.

## 6. Conclusions

In a clinical population where treatment effects can be difficult to sustain due to high relapse rates, service disengagement, and challenges with social functioning, the findings highlight the potential impact of HoryzonsCa for optimizing recovery in young people with FEP. Specifically, this study provides evidence on the acceptability of HoryzonsCa as participants expressed that HoryzonsCa had been helpful for recovery, expressed appreciation regarding its core elements (e.g., peer networking, therapeutic content, and moderation), and found it easy to navigate. On the other hand, not all participants were aware of important HoryzonsCa features, and some expressed concerns and suggestions regarding the platform. Overall, participants demonstrated a desire to see HoryzonsCa grow in scale, accessibility, and functionality.

## Figures and Tables

**Table 1:** Themes and subthemes.

Themes	Subthemes
Perceiving HoryzonsCa as helpful for recovery	Stress and anxiety coping Self-improvement and self-connectedness Applicable to everyday life and recovery Better informed about illness
Appreciating core intervention components	Connecting with peers Therapeutic content Moderation Ease and familiarity of use
Being unaware of HoryzonsCa’s features	Live chat Profile settings Communication tools
Expressing concerns, suggestions, and future directions	Lack of peer interaction Need for deeper content Wanting to connect more with moderators Desire for more professional design Future directions

## Data Availability

All the data that support the findings of this study are not publicly available due to their containing information that could compromise the privacy of research participants.

## Supplementary Materials

The following supporting information can be downloaded at: https://www.mdpi.com/1660-4601/20/9/5745/s1, Table S1. HoryzonsCa pilot study—focus group coding framework.
