# Supplementary material for: Experiences of a Digital Mental Health Intervention from the Perspectives of Young People Recovering from First-Episode Psychosis: A Focus Group Study
Source: Int J Environ Res Public Health. 2023 May 8;20(9):5745. doi: 10.3390/ijerph20095745 (PMC10177982; doi:10.3390/ijerph20095745)
Supplement: Supplementary file 1 [file ijerph-20-05745-s001.zip › ijerph-2217382-supplementary.pdf]

Table S1. HoryzonsCa pilot study—focus group coding framework.

Codes	Sub-Codes
1.General	1.1 General comments (e.g., likes and dislikes—related to Horyzons)<br>1.1.1 Expectations (in joining Horyzons)<br>1.1.1.1 To connect with others with similar experiences<br>1.1.1.2. To improve myself / life<br>1.1.1.3 Out of curiosity—not otherwise specified<br>1.1.1.4. Other<br>1.1.2. Impact of Horyzons on your daily life<br>1.1.3. Comments related to the future direction of Horyzons<br>1.2. General questions (related to Horyzons)<br>1.3 General comments and questions (unrelated to Horyzons)<br>1.4 General comments and questions (related to Horyzons research)<br>1.5 General neutral or unclear
2. Usefulness of platform features	2.1. Elements considered to be useful/helpful<br>2.1.1. What it was helpful for<br>2.1.1.1 Coping and recovery (e.g., anxiety, work, stress, and self-improvement/knowledge)<br>2.1.1.2. Feeling validated and understood<br>2.1.1.3. Skills acquisition<br>2.1.1.4. Finding employment<br>2.1.1.5. Other<br>2.1.2. Individually tailored experience<br>2.1.3. Community/peer network (ability to exchange re: hobbies, illness)<br>2.1.4. Content<br>2.1.5. Moderation<br>2.1.5.1. Peer Moderation<br>2.1.5.2. Clinical Moderation<br>2.1.6. Other platform features<br>2.2. Elements of concern and suggestions (not useful or missing)<br>2.2.1. Language concerns (i.e., French / English / bilingualism)<br>2.2.1.1. Suggestions (would improve)<br>2.2.2. Individually tailored experience concerns<br>2.2.2.1. Suggestions (would improve)<br>2.2.3. Community/peer network concerns<br>2.2.3.1. Suggestions (would improve)<br>2.2.4. Content concerns<br>2.2.4.1. Suggestions (would improve)<br>2.2.5. Clinical moderation concerns<br>2.2.5.1. Moderation suggestions (would improve)<br>2.2.5.2.1. Peer moderator concerns<br>2.2.5.2.1. Peer moderator suggestions (would improve)<br>2.2.5.3. Clinical moderator concerns<br>2.2.5.3.1. Clinical moderator suggestions (would improve)<br>2.2.6. Other platform feature concerns<br>2.2.6.1 Other platform suggestions (would improve)<br>2.3 Elements related to usefulness neutral—unclear<br>2.3.1. Individually tailored experience unclear<br>2.3.2. Community/peer network unclear<br>2.3.3. Content unclear<br>2.3.4. Clinical Moderation unclear

Lal, S.; Tobin, R.; Tremblay, S.; Gleeson, J.F.M.; D'Alfonso, S.; Etienne, G.; Joober, R.; Lepage, M.; Alvarez-Jimenez, M. Experiences of a Digital Mental Health Intervention from the Perspectives of Young People Recovering from First-Episode Psychosis: A Focus Group Study. *Int. J. Environ. Res. Public Health* **2023**, *20*, 5745. https://doi.org/10.3390/ijerph20095745

Table S1. *Cont.*

Codes	Sub-Codes
	2.3.4.1. Peer moderation unclear<br>2.3.4.2. Clinical moderator unclear<br>2.3.5. Other platform features unclear<br>2.4. Elements of the platform that have improved based on previous focus group feedback
3. Safety	3.1. Elements of the platform that enhance safety/support<br>3.2. Elements of concern regarding safety/support (not useful/missing)<br>3.2.1 Suggestions (would make it safer)<br>3.3. Safety/support questions<br>3.4. Safety/support neutral-unclear
4. Technical	4.1. Navigation<br>4.1.1 Likes<br>4.1.2 Dislikes or challenges<br>4.1.2.1. Suggestions (would improve)<br>4.2. Visual design<br>4.2.1 Likes<br>4.2.2 Dislikes<br>4.2.2.1. Suggestions (would improve)<br>4.3. Accessibility<br>4.3.1. Technical bugs<br>4.3.2. Device-related access<br>4.3.3. Other access-related comments<br>4.3.4. Accessibility suggestions (would improve)<br>4.4. Technical neutral-unclear
5. Participation	5.1. Actual use<br>5.1.1 Self<br>5.1.2 Others<br>5.2. Engagement (motivation)<br>5.2.1 Increases (self, others)<br>5.2.2 Limit/missing/would increase (self, others)<br>5.3. Willingness/desire to use Horyzons long term<br>5.4. Participation neutral-unclear
6. Orientation	6.1. Elements of orientation considered helpful<br>6.2. Orientation concerns (not useful/missing)<br>6.2.1 Suggestions (would improve)<br>6.3. Elements of the website that participants were unaware of<br>6.4. Orientation neutral-unclear
7. Meet-Up	7.1. Elements of the meet-up considered helpful<br>7.2. Meet-up concerns (not useful/missing)<br>7.2.1 Suggestions (would improve)<br>7.3 Meet-up neutral-unclear
8. Facilitator Summaries	8.1. Facilitator summaries of participant comments

Lal, S.; Tobin, R.; Tremblay, S.; Gleeson, J.F.M.; D'Alfonso, S.; Etienne, G.; Joober, R.; Lepage, M.; Alvarez-Jimenez, M. Experiences of a Digital Mental Health Intervention from the Perspectives of Young People Recovering from First-Episode Psychosis: A Focus Group Study. *Int. J. Environ. Res. Public Health* **2023**, *20*, 5745. https://doi.org/10.3390/ijerph20095745
